# Retraction: Decoration of graphene oxide nanosheets with carboxymethylcellulose hydrogel, silk fibroin and magnetic nanoparticles for biomedical and hyperthermia applications

**DOI:** 10.1039/d6na90013e

**Published:** 2026-02-19

**Authors:** Mostafa Ghafori Gorab, Hooman Aghamirza Moghim Aliabadi, Amir Kashtiaray, Mohammad Mahdavi, Milad Salimi Bani, Andisheh Etminan, Nabi Salehpour, Reza Eivazzadeh-Keihan, Ali Maleki

**Affiliations:** a Catalysts and Organic Synthesis Research Laboratory, Department of Chemistry, Iran University of Science and Technology Tehran 16846-13114 Iran maleki@iust.ac.ir reza.tab_chemist@yahoo.com +98-21-73021584 +98-21-73228313; b Advanced Chemical Studies Lab, Department of Chemistry, K. N. Toosi University of Technology Tehran Iran; c Endocrinology and Metabolism Research Center, Endocrinology and Metabolism Clinical Sciences Institute, Tehran University of Medical Sciences Tehran Iran; d Department of Biomedical Engineering, Faculty of Engineering, University of Isfahan Isfahan Iran; e School of Mechanical Engineering, Iran University of Science and Technology (IUST) Tehran Iran; f Department of Medical Biotechnology, Pasteur Institute of Iran Tehran Iran

## Abstract

Retraction of “Decoration of graphene oxide nanosheets with carboxymethylcellulose hydrogel, silk fibroin and magnetic nanoparticles for biomedical and hyperthermia applications” by Mostafa Ghafori Gorab *et al., Nanoscale Adv.*, 2023, **5**, 153–159, https://doi.org/10.1039/D2NA00394E.

The Royal Society of Chemistry hereby wholly retracts this *Nanoscale Advances* article due to concerns with the reliability of the data.

There are concerns with the hemolysis plate images in Fig. 5 being duplicated within the paper and the MTT plate images in Fig. 6 being duplicated in other articles by the same authors.^1,2^

The authors' response and data have been reviewed by an independent expert who has deemed them unsatisfactory.

Given the significance of these concerns, the editor has lost confidence that the findings presented in this paper are reliable.

This retraction supersedes the information provided in the Expression of Concern related to this article.

The authors were informed about the retraction of the article, Mohammad Mahdavi and Mostafa Ghafori Gorab have not agreed with the decision, the other authors have not indicated whether they agree with the decision to retract.

Signed: Jeremy Allen, Executive Editor, *Nanoscale Advances*

Date: 30th January 2026
